# Financing health care for adolescents: a necessary part of universal health coverage

**DOI:** 10.2471/BLT.14.139741

**Published:** 2014-10-27

**Authors:** Catriona Waddington, Claudia Sambo

**Affiliations:** a HLSP/Mott MacDonald, 10 Fleet Place, London EC4M 7RB, England.

Adolescents – defined by the World Health Organization (WHO) as persons aged 10–19 years – account for 1.2 billion or 18% of the world’s population.^1^ Adolescence is a crucial phase in human development, with rapid psychosocial and biological changes and it is often a period of experimentation and risk-taking. Health-related behaviours – such as patterns of alcohol use – affect physical and cognitive development, which can have an effect on long-term health. All these factors have implications for the types of health interventions that adolescents need.^2^ However, little is known about the impact of health financing choices on adolescents, a group rarely mentioned in the ongoing discussions about universal health coverage.

How health care is financed is an important element of achieving universal coverage. WHO’s health financing cube shows how to approach universal coverage (Fig. 1),^3^ which includes extension of coverage to more people, offering more services and reducing direct payments at the time of health service use. There are many ways to do this, but do they work for adolescents?

**Fig. 1:**
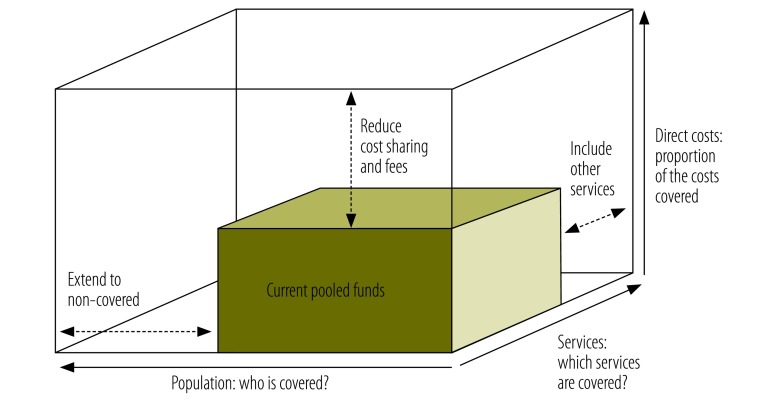
Three dimensions to consider when moving towards universal health coverage

Source: World Health Organization.^3^

Here we show how an explicit focus on adolescents can be used to examine the extent to which a health financing system meets the needs of this group. Questions need to be asked about adolescent membership in a pooled funding scheme, the fees they have to pay, and the availability of specific services.

## Adolescent health

Adolescent health is a significant global issue. Mortality for those aged 10–19 years is now greater than mortality in children aged 1–10 years. Improving adolescent health is important for achieving the health-related millennium development goals. In particular, lowering the rates of adolescent pregnancy is central to reducing both maternal and child mortality.^2^

It is impossible to quantify the extent of unmet need for adolescent health services. However information related to reproductive health, where more data is available, gives an idea of the issues. Adolescent girls who want to avoid pregnancy are more than twice as likely to have an unmet need for modern contraception as women aged 20–49 years. Each year, an estimated 6.1 million unintended pregnancies and 2.5 million unsafe abortions occur in adolescents in low- and middle-income countries.^4^

## Coverage

A pooled financing arrangement is when contributions from a sizeable group of people are brought together into a fund that pays for health services. In the health financing cube, the horizontal axis measures the proportion of the population covered by pooled health financing arrangements (Fig. 1). Poorer individuals are less likely to be covered than richer ones; adolescents in low- and middle-income countries are less likely to be covered than those in high-income countries.

Many governments have specific policies to improve coverage for children and adolescents. There are several ways in which health financing arrangements can be adapted to encourage use of health-care services by young people. In some countries governments pay insurance premiums for all children. This is the case in the Netherlands.^5^ Other governments pay premiums for some children; for example, for families below the poverty threshold in Andhra Pradesh, India.^6^ No co-payments are levied for children in Sweden or there are fewer co-payments than for adults, such as in Switzerland.^7^ In Egypt and in Viet Nam, promotion of insurance schemes through the school system has increased enrolment.^8^^,^^9^ *Health for the world’s adolescents* provides more information about ways in which adolescents are accounted for in various financing schemes.^2^

The age-range for adolescents who are entitled to benefits, such as exemptions from co-payment, varies from country to country: the most common cut-off age is 18 years, but it can be lower. Often older adolescents do not benefit from policies targeted to improve health care coverage for children. Even with specific pro-adolescent policies, there can be older adolescents who have either low or no incomes but are not covered by the financing arrangements.

## Costs for adolescents

The vertical axis of the financing cube tracks the proportion of costs covered by pooled funding, which affects both the levels and types of service used. 

Direct payments discourage adolescents from using health services. This is seen in countries at all stages of development. Direct payments also influence the type of services used by adolescents. In India and United Republic of Tanzania, for example, cost is a determinant in the choice of abortion provider and adolescent women are more likely to use the services of untrained, cheaper providers.^10^^,^^11^

## Service provision

The third axis of the financing cube tracks the proportion of services included in a financing arrangement. WHO has compiled a list of health-related interventions for adolescents in the areas of nutrition, substance use and tobacco control, mental health, sexual and reproductive health, maternal care, violence, injury prevention, human immunodeficiency virus infection, and vaccination. The services that are included in different financing schemes vary greatly. Many, such as care during childbirth, are generally provided as part of the health service as a whole. Some services, such as abortions, may be regarded as undesirable in general, while others, such as contraception, may be deemed unsuitable for an adolescent population. Some services are very specific and are likely to be included only if they are recognized as important in a particular country, e.g. opiate substitute therapy.

Decisions about services for adolescents are made by the agencies responsible for allocating the money in pooled health funds. Financing may not happen if adolescents are not seen as a priority in a contest for resources; if there is little awareness or information available about adolescents’ health needs; or if there is not a strong commitment to providing services related to potentially sensitive issues such as pre-marital sex, substance abuse or mental health.

Most adolescents are too young to vote and in many countries there is no organized lobbying on behalf of adolescents. This means that awareness-raising is often needed to attract support for expanding or improving services for adolescents. A report from the United Kingdom of Great Britain and Northern Ireland exploring the National Health Service in the context of services for young people called it a system which “forced care for children and young people into an unwinnable battle with adult care for influence on policy”.^12^ In contrast, services for the elderly were supported by powerful lobby groups.^13^ In the Republic of Moldova, there was limited interest in developing adolescent-friendly health services in the early 2000s. A small group of stakeholders (a few government officials, nongovernmental organizations and United Nations agencies) organized the first youth-friendly clinics. As the number of clinics increased, the interest in developing adolescent services widened to include the participation of adolescents themselves and the National Health Insurance Fund, which eventually started to finance some services previously paid for by external donors.^13^

Even if an adolescent is entitled to access services financed with pooled funds, these services may not be appropriate or socially acceptable to them. Adolescents will use services less if they feel that health workers are judgmental or will not maintain confidentiality. 

## Towards adolescent-proof health services

The challenges of making universal health coverage a reality for adolescents are in some ways the same as for other age groups: society’s poor and marginalized people are the hardest to provide for. Adolescent health care brings additional challenges, since adolescents are more likely to have less access to cash and specific needs for confidential health care and because adolescent health care is often a relatively low priority. Adolescents are neither children nor adults; two groups that health systems clearly distinguish. They therefore risk falling into a policy gap where their specific needs are overlooked. 

There are several types of health financing arrangement for universal health coverage and several ways to ensure adolescent coverage. The challenge is to develop policies and practices that meet adolescents’ specific needs. Looking at health financing arrangements, policies and services through an adolescent’s perspective may be a starting point. This involves three questions. First, are adolescents adequately covered by a pooled financing arrangement (insurance- or tax-based)? If not, there may need to be a focus on increasing overall coverage and/or on measures that particularly target adolescents, including older adolescents. Second, do adolescents have to pay fees to use essential services, and if so, what is the impact of fees on their use of services? If this is a problem, exemptions could be a short-term measure. Third, are the services that are appropriate for adolescents covered by pooled financing arrangements? If not, it may be that the services do not exist in the country or that the financing scheme chooses not to fund them. The question can trigger discussions about how to encourage adolescent services, for example by recognizing that pregnant adolescents may need additional support and that this comes at a cost.

The needs of adolescents are complex and cannot be summarized in a shortlist of things to provide. These questions around the three dimensions of the health financing cube can be used to start thinking about services and health financing arrangements from an adolescent’s perspective. The framework is relevant to a variety of stakeholders, from ministries of health, social security agencies and insurance organizations, to clinicians, parents and adolescents themselves.
